# Alginate oligosaccharides derived from tropical brown seaweeds as sustainable alternatives to antibiotic growth promoters in poultry nutrition: Functional mechanisms and production perspectives

**DOI:** 10.14202/vetworld.2026.224-263

**Published:** 2026-01-20

**Authors:** Sepri Reski, Maria Endo Mahata, Yose Rizal, Yelsi Listiana Dewi

**Affiliations:** 1 Department of Nutrition Science and Feed Technology, Faculty of Animal Science, Universitas Andalas, Padang, 25163, West Sumatra, Indonesia; 2 Research Center of Animal Husbandry, Research Organization for Agriculture and Food, National Research and Innovation Agency (BRIN), Jl. Raya Jakarta-Bogor, Cibinong 16915, West Java, Indonesia

**Keywords:** alginate oligosaccharides, antibiotic alternatives, gut health, poultry nutrition, prebiotics, seaweed bioactives, sustainable feed additives, tropical seaweed

## Abstract

The global restriction and withdrawal of antibiotic growth promoters (AGPs) in poultry production have accelerated the search for natural, safe, and sustainable feed additives that maintain bird health and productivity. Alginate oligosaccharides (AOS), derived from the depolymerization of alginate present in brown seaweeds, have gained increasing attention due to their multifunctional biological properties, including prebiotic, immunomodulatory, antioxidant, and antimicrobial activities. Although most available research has focused on alginate sources from temperate seaweeds, tropical brown seaweeds such as *Sargassum* and *Turbinaria* are abundant, renewable, and rich in alginate, particularly in Southeast Asia, making them attractive and underutilized resources for the development of functional feed additives. This review summarizes current knowledge on the biodiversity of tropical brown seaweeds, alginate extraction and depolymerization techniques suitable for feed-grade AOS production, and the physicochemical properties that influence their functionality in poultry nutrition. Emphasis is placed on AOS behavior in the poultry gastrointestinal tract, including resistance to enzymatic digestion, fermentation by beneficial microbiota, and stimulation of short-chain fatty acid production. Evidence from experimental studies indicates that dietary AOS supplementation improves gut morphology, enhances microbial balance, strengthens intestinal barrier function, and modulates immune responses. These effects are consistently associated with improved growth performance, feed efficiency, egg production, and antioxidant status, with outcomes comparable to or exceeding those achieved using AGPs. The review also highlights emerging processing strategies, such as low-energy extraction and encapsulation technologies, that enhance AOS stability and bioavailability during feed manufacturing. Overall, tropical seaweed-derived AOS represent a promising, sustainable alternative to AGPs in poultry systems, supporting productivity while addressing antimicrobial resistance and environmental sustainability concerns. Further large-scale field studies and optimization of dosage and formulation strategies are recommended to facilitate commercial adoption.

## INTRODUCTION

The global livestock and poultry sectors are undergoing a fundamental shift in feed management, driven largely by increasing concerns surrounding the use of antibiotic growth promoters (AGPs). Robust scientific evidence has established a clear link between the non-therapeutic use of antibiotics in animal production and the emergence of antimicrobial resistance in human pathogens. In response, several national and international regulatory authorities, including those in the European Union and the United States, have implemented stringent bans and restrictions on the inclusion of AGPs in animal feed [1–3]. These regulatory actions are underpinned by public health priorities and heightened consumer awareness regarding antibiotic residues in animal-derived food products [4, 5]. Consequently, the progressive withdrawal of AGPs has intensified the need to identify effective alternatives capable of sustaining animal productivity and health.

This transition has stimulated extensive research into natural, safe, and sustainable feed additives that can enhance growth performance and immune function without the adverse consequences associated with antibiotics. Various alternatives, including probiotics, phytogenic compounds, and prebiotics, have been proposed and evaluated as potential substitutes for AGPs [6–8]. These strategies aim not only to preserve animal performance but also to meet consumer demand for antibiotic-free animal products while reducing risks related to food safety and public health [9–11].

Within this context, alginate and its derivatives, particularly alginate oligosaccharides (AOS), have attracted increasing attention as promising functional feed ingredients. Alginate is a naturally occurring polysaccharide composed of β-D-mannuronic acid and α-L-guluronic acid residues arranged in distinct block structures that determine its physicochemical properties [12]. Key functional characteristics of alginate, including gel formation, viscosity, and solubility, are influenced by molecular weight and the mannuronic-to-guluronic acid (M/G) ratio, which vary according to species origin, extraction methods, and environmental conditions [13].

Alginate can be depolymerized through enzymatic or chemical processes to generate AOS, oligomers typically consisting of 2–25 monomer units [14]. Compared with native alginate polymers, AOS exhibit greater solubility, lower viscosity, and improved bioavailability, making them particularly suitable for inclusion in animal diets [15, 16]. Numerous studies have demonstrated that AOS possess diverse biological activities, including immunomodulatory, antimicrobial, antioxidant, and prebiotic effects [17]. These multifunctional properties have driven their evaluation as feed additives, especially for monogastric animals such as poultry and swine.

In poultry nutrition, AOS have shown considerable potential to improve gut health and overall performance. Enzymatically produced AOS have been reported to enhance broiler growth by modulating the composition and activity of the intestinal microbiota [18]. Similarly, dietary AOS supplementation has been shown to improve protein and fat digestibility in piglets, thereby enhancing growth performance [19]. These outcomes align with global efforts to reduce antibiotic usage in animal agriculture while promoting gut health through nutritional interventions [15].

The biological efficacy of AOS is closely linked to its molecular structure. Factors such as degree of polymerization (DP), monomer sequence, and the presence of unsaturated terminal residues significantly influence their interactions with gut microbiota and immune cells [20]. Short-chain AOS exhibit stronger immunostimulatory activity than longer-chain counterparts [20]. In vitro studies further indicate that AOS can regulate cytokine production and activate key immune signaling pathways, including nuclear factor-κB (NF-κB) and mitogen-activated protein kinases (MAPKs), which are central to immune modulation [17]. In addition, AOS demonstrate antimicrobial activity against a range of bacterial species, including drug-resistant strains [21], and exhibit prebiotic effects by selectively increasing populations of beneficial bacteria such as *Bifidobacterium* and *Lactobacillus* without inducing adverse gastrointestinal effects [22, 23].

Despite these promising attributes, the majority of existing research and commercial applications have focused on AOS derived from temperate brown seaweeds, including *Laminaria*, *Ascophyllum*, and *Macrocystis*. In contrast, tropical brown seaweeds, particularly species within the *Sargassum* and *Turbinaria* genera, remain underexplored, despite their abundance in countries such as Indonesia. Favorable environmental conditions, including high solar radiation, warm seawater temperatures, and nutrient-rich coastal ecosystems, support extensive growth of these species throughout the Indonesian archipelago [24, 25]. These seaweeds exhibit high biomass productivity and substantial alginate content, making them attractive and sustainable feedstocks for AOS production [26, 27].

The exploration of AOS derived from tropical seaweeds is therefore of considerable significance. These bioactive compounds offer a sustainable, environmentally friendly alternative to conventional feed additives, as seaweed resources are renewable [28]. Incorporation of AOS into poultry diets has the potential to enhance immune competence, improve disease resistance, and reduce mortality under intensive production systems [29, 30]. Moreover, improved feed efficiency associated with AOS supplementation may enable more effective conversion of feed into poultry biomass, thereby reducing production costs and increasing economic returns for producers [28]. Importantly, this approach aligns with global initiatives to reduce reliance on synthetic additives and antibiotics, address antimicrobial resistance concerns, and meet consumer expectations for residue-free poultry products [30].

The economic implications of AGP bans are substantial, with significant market impacts reported in the poultry sector as regulatory frameworks increasingly prioritize animal health and food safety [31]. Global seaweed production is estimated at approximately 30 million tons annually, underscoring the availability of largely untapped alginate sources such as *Sargassum* and *Turbinaria*. Nevertheless, despite extensive research on temperate alginate sources, data on tropical species remain limited, particularly in the context of poultry nutrition [31]. *Sargassum* species are widely distributed along Indonesian coastlines and contain appreciable levels of protein (6.21%–8.54%) and polysaccharides, including alginate [27]. Similarly, *Turbinaria* species are known for their high sodium alginate yield, with alginate content reaching up to 40% of dry-weight, highlighting their potential as renewable raw materials for the production of functional feed additives [25].

The use of tropical seaweed-derived AOS in poultry feed aligns with broader sustainability objectives and offers a viable response to the challenges posed by AGP withdrawal. These compounds can effectively mimic or replace AGPs by enhancing nutrient absorption, modulating immune responses, and supporting a balanced gut microbiome. Their multifunctional properties, renewable origin, and cost-effectiveness further strengthen their suitability for industrial feed formulation [26].

To maximize the benefits of AOS, several practical considerations must be addressed. Optimization of extraction and formulation processes using locally available seaweed species is essential to ensure cost-effectiveness [32]. Attention to alginate grade and purity is critical, as contaminants may compromise the biological efficacy of the resulting oligosaccharides. Additionally, combining AOS with complementary additives such as probiotics or other prebiotics may enhance their functional effects on gut health and immunity [33]. Dosing strategies should also be tailored according to poultry species, age, and environmental stressors, as variable responses to AOS have been reported across different production contexts [34]. Continuous monitoring of health and performance indicators will be necessary to evaluate efficacy and guide dietary adjustments.

Current evidence indicates that AOS are generally regarded as safe and non-toxic for poultry. Their high solubility and non-immunogenic nature support their suitability for dietary inclusion without associated toxicity risks [35]. The antioxidant properties of AOS further contribute to their safety profile by supporting physiological resilience under stress conditions [36]. Moreover, their demonstrated biocompatibility suggests that AOS can be incorporated into poultry diets without adverse effects on animal health [37]. Although consensus on maximum safe inclusion levels has yet to be established, available studies indicate favorable tolerance profiles. Notably, AOS do not exert direct inhibitory effects on pathogens such as *Salmonella enterica* serovar Typhimurium, suggesting that they modulate gut microbiota without disrupting beneficial microbial populations [38].

Despite growing interest in AOS as functional feed additives, several critical research gaps remain that limit their broader application in poultry nutrition. First, the majority of existing studies and commercial developments have focused on AOS derived from temperate brown seaweeds such as *Laminaria*, *Ascophyllum*, and *Macrocystis*, while tropical brown seaweeds, including *Sargassum* and *Turbinaria*, remain comparatively underexplored. This imbalance persists despite the high abundance, rapid biomass turnover, and substantial alginate content of tropical species, particularly in Southeast Asia. Consequently, knowledge regarding the chemical composition, mannuronic-to-guluronic acid ratios, seasonal variability, and functional characteristics of tropical seaweed-derived alginates remains fragmented and insufficiently synthesized.

Second, although numerous studies have demonstrated beneficial effects of AOS on gut health, immune modulation, and performance in poultry, results are often inconsistent due to differences in AOS molecular characteristics, extraction methods, degrees of polymerization, and inclusion levels. Limited attention has been given to systematically linking AOS structural properties with their biological functions in the avian gastrointestinal tract (GIT). Furthermore, mechanistic insights into AOS metabolism, microbial fermentation dynamics, short-chain fatty acid production, and immune signaling pathways in poultry are still incomplete, with much of the available evidence extrapolated from mammalian or in vitro models rather than avian-specific systems.

Third, practical and translational aspects remain inadequately addressed. Comparative evaluations of extraction and depolymerization techniques suitable for feed-grade AOS production from tropical seaweeds are scarce, as are assessments of formulation stability, encapsulation strategies, dosage optimization, and economic feasibility under commercial poultry production conditions. Additionally, while AOS are generally regarded as safe, there is no consensus on optimal inclusion levels, long-term safety, or interactions with other feed additives such as probiotics and phytogenics. Collectively, these gaps hinder the effective integration of tropical seaweed-derived AOS into sustainable, antibiotic-free poultry feeding programs.

In light of these gaps, the present review aims to provide a comprehensive and integrative evaluation of AOS derived from tropical brown seaweeds as functional feed additives in poultry nutrition. Specifically, this review seeks to (i) summarize the biodiversity, availability, and alginate characteristics of major tropical brown seaweeds with relevance to AOS production; (ii) critically examine current extraction, depolymerization, and formulation strategies for producing feed-grade AOS, with emphasis on sustainability and scalability; (iii) elucidate the physicochemical properties and gastrointestinal behavior of AOS in poultry, including digestion resistance, microbial fermentation, and metabolic fate; and (iv) synthesize evidence on the functional effects of AOS on poultry growth performance, gut health, immune responses, and antioxidant status in comparison with conventional AGPs.

In addition, this review aims to identify key limitations in current knowledge and highlight future research priorities, including dose optimization, structure–function relationships, synergistic feed formulations, and large-scale field validation. By consolidating dispersed evidence and emphasizing tropical seaweed resources, this review intends to support the development of sustainable, effective, and economically viable AOS-based strategies that contribute to antibiotic-free poultry production and global efforts to mitigate antimicrobial resistance.

## METHODOLOGY

This narrative review was designed to systematically evaluate and synthesize current evidence on the use of AOS derived from tropical seaweeds as functional feed additives in poultry nutrition. A structured and transparent approach was adopted to identify, select, analyze, and integrate relevant scientific literature.

### Literature search strategy

A comprehensive literature search was conducted using major electronic databases, including PubMed, ScienceDirect, and Google Scholar. Relevant studies were identified using combinations of the following keywords: *tropical seaweed alginate*, *alginate oligosaccharides*, *poultry feed additives*, and *alternatives to AGPs*. Reference lists of selected articles were also screened to identify additional relevant publications.

### Eligibility criteria

Peer-reviewed articles published within the last 10 years were considered eligible for inclusion. Studies were selected if they addressed one or more of the following aspects: chemical characteristics of alginate and AOS, extraction and depolymerization methods, physicochemical properties, biological activities, and applications of AOS in poultry nutrition. Only articles published in English and available as full texts were included. Conference abstracts, non-peer-reviewed reports, and studies lacking sufficient methodological detail were excluded.

### Data extraction and evidence synthesis

Data were systematically extracted from eligible studies, including information on seaweed species used, alginate extraction techniques, AOS production methods, molecular and physicochemical properties, and reported biological effects in poultry. Particular emphasis was placed on studies evaluating growth performance, gut health, immune modulation, and nutrient utilization. Where applicable, findings from studies comparing AOS supplementation with conventional AGPs were highlighted to assess their relative efficacy.

### Assessment of chemical and functional characteristics

The review critically examined the chemical composition of alginate obtained from tropical seaweeds, with specific focus on the mannuronic-to-guluronic acid (M/G) ratio, DP, and their influence on the bioactivity and functional performance of AOS. The prebiotic potential of AOS, including effects on gut microbiota composition and nutrient absorption, was also evaluated based on available experimental evidence.

### Evaluation of poultry performance outcomes

Poultry performance parameters assessed in this review included average daily gain (ADG), feed conversion ratio (FCR), egg production, immune response indicators, and measures of gut health. Outcomes from AOS-supplemented diets were compared with those reported for AGP-based feeding strategies to determine whether AOS could provide comparable or superior benefits under different production conditions.

### Quality assessment of included studies

The quality of included studies was assessed based on methodological rigor, sample size, experimental design, and consistency of reporting. Meta-analyses and systematic studies were prioritized when available. Studies exhibiting major methodological limitations or insufficient data were excluded to ensure the reliability and scientific robustness of the synthesized evidence.

### Alginate and AOS: Structural overview

AOS are derived from alginate, a naturally occurring polysaccharide primarily located in the cell walls of brown seaweeds. AOS represent depolymerized fragments of alginate composed of linear chains containing 2–25 monomer units. These monomers consist of two uronic acids, α-L-guluronic acid (G) and β-D-mannuronic acid (M)—linked by 1,4-glycosidic bonds, forming linear polymers whose arrangement governs their physicochemical and biological properties [15].

Structurally, alginate and its oligosaccharides are characterized by homopolymeric blocks of mannuronate (polyM), guluronate (polyG), or heteropolymeric sequences (polyMG or polyGM) [39]. This diversity arises from biological synthesis in algae or enzymatic depolymerization mediated by alginate lyases, which selectively cleave glycosidic bonds within alginate chains [40]. AOS retain key functional properties of alginate, including high solubility and low viscosity, while lacking the strong gelling capacity typical of high-molecular-weight alginates [15].

## PHYSICOCHEMICAL AND BIOLOGICAL PROPERTIES OF AOS

AOS exhibit high water solubility and absorption capacity, enabling their application in food, biomedical, and animal nutrition systems. Functionally, AOS act as prebiotics that stimulate beneficial gut microbiota and possess antioxidant activity capable of scavenging free radicals [41]. Their biological efficacy is strongly influenced by molecular weight and composition, with lower-molecular-weight AOS displaying enhanced bioactivity compared with intact alginate polymers.

The DP, which typically ranges from 2 to 25 depending on the depolymerization process, plays a crucial role in determining antioxidant, immunomodulatory, and prebiotic properties [42, 43]. Variations in the M/G ratio further influence immune responses through modulation of immune cell signaling pathways, including toll-like receptor 4 (TLR4) activation and subsequent cytokine production [17]. Sulfated AOS exhibit stronger antioxidant activity than non-sulfated forms, with optimal biological activity observed at specific DP ranges [42]. Tropical seaweed-derived AOS may exhibit enhanced immunomodulatory potential due to higher guluronic acid content and the presence of unsaturated uronic acid termini.

## MACROALGAL BIODIVERSITY IN TROPICAL REGIONS

Tropical brown seaweeds represent a highly diverse and ecologically significant group of macroalgae distributed across equatorial regions, particularly Southeast Asia and tropical Africa. Warm temperatures, high solar radiation, and nutrient-rich coastal waters support continuous growth throughout the year [44, 45]. Dominant genera include *Sargassum*, *Turbinaria*, *Padina*, and *Hormophysa*, each contributing uniquely to ecosystem structure and productivity.

Species such as *Sargassum* and *Turbinaria* are highly productive biomass sources and are rich in bioactive compounds with applications in food, feed, and biotechnology [46]. Beyond their industrial value, these seaweeds play essential ecological roles, supporting marine biodiversity and maintaining coastal ecosystem stability [47]. Tropical species often exhibit higher biomass yields and distinct biochemical profiles compared with temperate counterparts, including greater accumulation of polysaccharides and beneficial lipids [48, 49]. Differences in M/G ratios between tropical and temperate habitats further influence alginate quality and functionality [50].

## INDONESIA AS A KEY RESOURCE FOR TROPICAL ALGINATE

Indonesia possesses some of the most productive coastal ecosystems globally, including coral reefs, mangroves, and seagrass beds that support extensive seaweed growth [47]. The biotechnological exploitation of alginate from locally abundant seaweeds presents opportunities for novel applications in animal feed, nutraceuticals, and pharmaceuticals [51]. Favorable environmental conditions and ecosystem resilience enable sustained utilization of algal biomass for functional feed ingredients, often with fewer constraints than those encountered in temperate regions [52].

Morphologically, *Sargassum* species are characterized by branched thalli, gas-filled vesicles for buoyancy, and robust holdfasts that facilitate colonization of intertidal and subtidal zones [44, 45]. *Turbinaria* species possess thick, leathery fronds adapted to high-energy coastal environments, while *Padina* exhibits calcified blades that deter herbivory. *Hormophysa* is distinguished by smooth, undulated fronds, illustrating the structural diversity of tropical brown seaweeds [44].

## ECOLOGICAL AND GEOGRAPHIC DISTRIBUTION

Tropical brown seaweeds serve as primary producers, sediment stabilizers, and habitat formers. *Sargassum* beds create complex three-dimensional structures that provide shelter and feeding grounds for juvenile fish and invertebrates [53]. *Turbinaria* contributes to sediment stabilization and nutrient cycling, enhancing the resilience of adjacent coral reef and seagrass ecosystems [45].

Geographically, *Sargassum* dominates Indonesian coastlines, particularly along Java, supported by nutrient influxes and favorable tidal regimes [44]. In Malaysia and the Philippines, *Sargassum* and *Turbinaria* thrive in coastal and lagoon systems, while West African coastlines also support substantial biomass due to stable currents and upwelling [45, 53]. Continuous tropical light and warmth enable uninterrupted photosynthesis, supporting frequent harvesting and positioning these seaweeds as sustainable alginate sources.

## ECONOMIC RELEVANCE AND MARKET TRENDS

The incorporation of functional feed additives has demonstrated improvements in poultry performance while addressing economic and consumer safety concerns. Alternatives to AGPs, including prebiotics and probiotics, have improved feed efficiency, immune responses, and product safety [54–56]. Economic assessments consistently report favorable cost–benefit ratios, supporting sustainable poultry production practices [57].

Commercial AOS-based feed additives have recently entered the market, particularly in tropical regions. Market analyses indicate strong growth in plant-based feed additives driven by antimicrobial regulations, with seaweed-derived products showing a compound annual growth rate exceeding 10% [58, 59]. Southeast Asian countries, including Indonesia, Malaysia, and the Philippines, have emerged as leaders in developing locally sourced alginate feed supplements [59]. The market for AOS as feed additives is surging, propelled by the poultry industry’s need for sustainable alternatives to traditional feed additives amid escalating feed prices and increasing concerns over feed safety issues, particularly related to mycotoxins commonly found in poultry feeds in tropical regions [60].

Research conducted by Dewi *et al*. [61] demonstrates that the fermentation of *Sargassum binderi* can enhance its nutritional profile for laying hens, although the specific reduction of alginate content should be verified with further studies. Collaborations between Malaysia aquaculture firm and research institutions are exploring the efficacy of AOS as functional feed additives to promote gut health and improve in poultry [62].

## REGULATORY FRAMEWORK AND ADOPTION CHALLENGES

In the European Union, feed additives are regulated by the European Food Safety Authority, which requires comprehensive safety evaluations, including contaminant risk assessment [63]. Similar regulatory oversight is exercised by the United States Food and Drug Administration [64]. In the ASEAN region, regulatory frameworks vary among member states, often relying on evidence generated in other regions, underscoring the need for poultry-specific efficacy and safety data for AOS adoption [65].

### Sustainability and circular bioeconomy perspectives

Integrating alginate production with aquaculture and poultry systems supports multiple Sustainable Development Goals, including SDG 2 (zero hunger), SDG 12 (responsible consumption), and SDG 14 (life below water) [66–69]. Circular bioeconomy models enable by-products from one sector to support another, enhancing resource efficiency and reducing waste [70, 71]. While tropical seaweeds offer high renewability and carbon sequestration potential, responsible harvesting and farming practices are essential to prevent ecosystem disruption [72–74]. Incorporation of seaweed-derived AOS into poultry diets further enhances sustainability by improving feed efficiency, reducing reliance on synthetic additives, and lowering greenhouse gas emissions [75].

### Influence of seasonal and environmental factors

Seasonal variation and geographical location strongly influence the biomass yield and alginate composition of tropical brown seaweeds. Environmental parameters such as temperature, salinity, light intensity, and nutrient availability regulate metabolic activity and polysaccharide biosynthesis in these algae. For example, *Turbinaria conoides* has been reported to exhibit higher alginate yields during pre-monsoon periods, reflecting favorable growth and metabolic conditions during this season [76]. Such environmental fluctuations result in measurable changes in both total biomass production and the physicochemical characteristics of extracted alginate.

Quantitative data summarizing alginate content, mannuronic-to-guluronic acid (M/G) ratios, and seasonal variability among different seaweed species are presented in Table 1 [15, 16, 77–79]. While temperate species such as *Laminaria* and *Ascophyllum* generally exhibit higher alginate content with pronounced seasonal variation, tropical species, including *Sargassum*, consistently provide moderate yet valuable alginate yields, supporting their suitability as sustainable biomass sources in tropical regions.

**Table 1:** Alginate content, mannuronic-to-guluronic acid (M/G) ratio, and key compositional characteristics of selected temperate and tropical brown seaweeds used for alginate and alginate oligosaccharide production.

Seaweed species	Alginate content (% dry weight)	M/G ratio range	Seasonal variability	Reference
*Laminaria* spp.	25-30	1.5-2.5	Significant variations noted between seasons	[15, 16]
*Ascophyllum nodosum*	30-40	1.0-2.0	Peak harvesting typically occurs in late spring and early summer	[77]
*Turbinaria murayana*	20-25	1.2-2.0	Exhibits fluctuations based on water temperature and nutrient availability	[78]
*Sargassum* spp.	12.20	1.0-1.5	Seasonal changes are prominent, especially during peak growth seasons	[79]

Values are reported on a dry-weight basis. M/G ratio indicates the molar ratio of β-D-mannuronic acid to α-L-guluronic acid residues. Variations reflect differences in species, geographic origin, season, and extraction conditions.

## ALGINATE CONTENT AND FUNCTIONAL QUALITY

Brown seaweeds are recognized for their high alginate content, which may constitute up to 40% of dry-weight in certain species [80]. However, alginate functionality is determined not only by total yield but also by its monomeric composition, particularly the M/G ratio. This ratio governs key functional properties such as gel strength, viscosity, and elasticity. Higher guluronic acid content enhances calcium-mediated crosslinking, resulting in stronger and more rigid gels, whereas higher mannuronic acid proportions confer greater flexibility and elasticity [12].

Studies indicate that tropical seaweeds typically exhibit M/G ratios ranging from 0.43 to 2.52, with many species clustering around intermediate values of 1.0–1.2 [81]. These balanced ratios are especially advantageous for producing bioactive AOS, as moderately flexible polymer structures are more susceptible to enzymatic depolymerization and microbial fermentation within the GIT, thereby enhancing their functional potential in animal nutrition [82].

## EFFECTS OF EXTRACTION AND PROCESSING METHODS

Alginate yield and quality are also strongly influenced by extraction and processing conditions. Alkaline extraction, the most commonly employed method, is sensitive to variables such as pH, temperature, extraction time, and the use of chelating agents, all of which affect yield, purity, and molecular weight [83]. Notably, alginate extracted from tropical seaweeds often exhibits lower viscosity and molecular weight compared with alginate from temperate species such as *Ascophyllum nodosum*. These characteristics may favor subsequent depolymerization into AOS, enhancing suitability for feed-grade applications [15].

Comparative compositional data for alginate extracted from various seaweed species are summarized in Table 2 [84–91]. Reported M/G ratios indicate that *Sargassum* spp. typically yield alginates with ratios ranging from 0.80 to 1.10, whereas species such as *Fucus vesiculosus* tend to exhibit higher mannuronic acid content, resulting in greater elasticity [86, 88, 91]. These compositional differences directly influence alginate performance across food, nutraceutical, and feed applications.

**Table 2:** Physicochemical properties of alginate extracted from selected brown seaweed species using different extraction methods.

Seaweed species	Specific alginate yield (%)	M/G ratio	Purity level (%)	Extraction condition	Reference
*Sargassum* spp.	Varies; high yield reported around 25-30%	0.80–1.10	85.90%	Alkali extraction methods at varying temperatures	[84–86]
*Sargassum natans*	Approx. 20%	0.88	High purity confirmed	Alkali extraction and precipitation with ethanol	[87, 88]
*Lessonia flavicans*	Easonal variation affects yield	Not specified	Not specified	Harvest timing effects composition	[89]
*Rugulopteryx okamurae*	High yield (specific values not stated)	0.88	Not specified	RO extraction method	[88]
*Durvillaea antartica*	Yield can vary; indicative high levels	~1.00	Variable purity	Seasonal extraction impact	[85]
*Sargassum turbinarioides*	Yield not provided	~0.87	~90%	Commonly alkali extracted	[90]
*Fucus vesiculosus*	Yield variability reported; high; >20%	>1.00	Good purity; close to commercial standards	Viscosity and purity depend on methods	[91]

DP = Degree of polymerization, M/G = Mannuronic-to-guluronic acid ratio. Reported values depend on extraction pH, temperature, duration, and purification procedures.

## MICROBIAL AND BACTERIAL SOURCES OF ALGINATE

### Microbial alginate biosynthesis

In addition to macroalgal sources, microbial production of alginate represents an alternative pathway, particularly for applications requiring high purity and precise compositional control. Bacterial species such as *Azotobacter vinelandii* and *Pseudomonas balinensis*, including strains isolated from tropical environments, are capable of synthesizing alginate via regulated biosynthetic pathways [92, 93].

Microbial alginates share structural similarity with macroalgal alginates, consisting of linear chains of β-D-mannuronic and α-L-guluronic acids. However, they often exhibit higher degrees of acetylation and more uniform molecular weight distributions due to tightly controlled fermentation conditions [94]. Factors such as oxygen availability during *A. vinelandii* cultivation significantly influence polymer length and functional group composition, enabling fine-tuning of alginate properties [94].

### Advantages of microbial production systems

Microbial alginate extraction is typically conducted under mild processing conditions, offering advantages in reproducibility, environmental sustainability, and product consistency [95, 96]. Unlike macroalgal extraction, which depends on seasonal biomass availability and may involve harsh chemical treatments, microbial fermentation allows year-round production and precise control over alginate characteristics. Although total yields from microbial sources are generally lower than those from macroalgae, this approach enables the production of alginate tailored for specialized applications, including biomedical uses and precursors for AOS with defined bioactivities.

### Enzymatic depolymerization and AOS production

Targeted enzymatic hydrolysis using alginate lyases, particularly enzymes belonging to the polysaccharide lyase family 7 (PL7), facilitates controlled depolymerization of both macroalgal and microbial alginate into low-degree-of-polymerization oligosaccharides [97]. PL7 lyases exhibit activity against both polyM and polyG blocks, generating AOS with diverse structural profiles. Enzymes such as PsMan8A display substrate specificity toward polyM regions, enabling tailored AOS production [98].

Importantly, enzymatically generated AOS preserve unsaturated uronic acid termini and functional groups associated with enhanced immunomodulatory and prebiotic activities [17]. This mild and environmentally friendly approach supports sustainable AOS production for feed applications [14].

### Advances in enzyme technology and fermentative pathways

Recent research has focused on developing cold-adapted and engineered alginate lyases capable of operating efficiently at lower temperatures, thereby reducing energy requirements and reliance on high-temperature processing [99]. These innovations improve biomass utilization efficiency and align with biorefinery principles.

Fermentative production of AOS using specific microbial strains has also emerged as a promising alternative. Pathways involving indigenous gut microbiota or selected fermentative organisms have demonstrated potential for optimizing AOS yields, particularly from tropical alginate sources [100]. Such microbial-based synthesis offers a sustainable alternative to chemical depolymerization methods within tropical agricultural systems.

### Comparative yield and economic considerations

Production pathways for alginate differ markedly between macroalgal extraction and microbial fermentation. Macroalgal extraction from brown seaweeds such as *Laminaria* typically yields approximately 1% alginate on a dry-weight basis under sustainable harvesting conditions. However, costs associated with collection, processing, and seasonal variability can limit economic feasibility, with raw alginate prices ranging from approximately USD 3–5 per kg [78].

In contrast, microbial production systems using *Pseudomonas* and *Azotobacter* species can yield alginate equivalent to 10%–15% of microbial biomass under controlled conditions. Estimated production costs of USD 2–4 per kg reflect advantages of scalability, continuous production, and reduced environmental impact [15].

### Integrated production perspectives

In summary, tropical macroalgae and microbial systems represent complementary platforms for alginate and AOS production. Macroalgae provide abundant, renewable biomass, while microbial systems offer precision, consistency, and process control. Integration of these approaches may optimize yield, quality, and application diversity, supporting broader adoption of AOS as natural alternatives in poultry nutrition.

**Figure 1:**
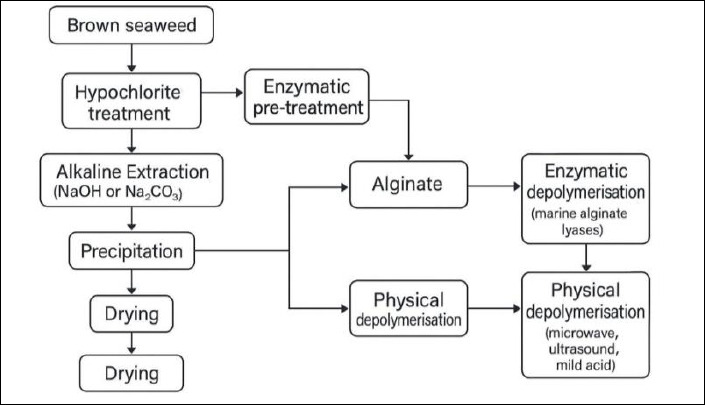
Schematic overview of alginate extraction from brown seaweed and subsequent depolymerisation pathways. The process includes hypochlorite treatment, alkaline extraction, precipitation, and drying to obtain alginate, followed by depolymerisation using enzymatic (marine alginate lyases) or physical methods (microwave, ultrasound, or mild acid).

Key characteristics of tropical brown seaweeds suitable for alginate production are summarized in Table 3 [12, 28, 29, 44, 45, 53], including morphological traits, geographical distribution, alginate content, M/G ratios, and ecological roles of *Sargassum*, *Turbinaria*, *Padina*, and *Hormophysa*. The overall workflow for tropical alginate extraction and AOS production is illustrated in Figure 1, depicting sequential steps from seaweed harvesting and drying through alginate extraction, purification, depolymerization, and final product processing.

**Table 3:** Major tropical brown seaweed genera, geographic distribution, alginate yield, and ecological characteristics relevant to sustainable alginate sourcing.

Genus	Morphological features	Geographic distribution	Alginate content (% DW)	Typical M/G Ratio	Ecological role	Reference
*Sargassum*	Branched thalli, air vesicles, strong holdfasts	Indonesia (Java, Sulawesi), Malaysia, Philippines, West Africa	Up to 40%	~1.0–1.2	Habitat formation, coastal protection, biodiversity hub	[44, 45, 53]
*Turbinaria*	Leathery fronds, convoluted thick blades	Malaysia, Indonesia, Philippines, West Africa	~30%–35%	~0.9–1.1	Sediment stabilization, nutrient cycling	[44, 45, 53]
*Padina*	Calcified blades, fan-shaped thallus	Philippines, Malaysia, Indonesia	~20%–30%	Variable (~0.8–1.3)	Structural habitat, herbivory deterrence	[28, 29]
*Hormophysa*	Smooth, undulated thallus	Southeast Asia, tropical Africa	~25%–30%	~1.0	Biofilm substrate, habitat complexity	[44, 45]
Laminaria*	Long flat blades, large size	Temperate coasts (comparison species)	17%–45%	High G (>1.5)	Commercial alginate production, strong gel-forming	[12]

* *Laminaria* is included for reference as a temperate genus often used in commercial alginate production. Alginate yield is expressed as a percentage of dry biomass. Geographic distribution reflects the dominant coastal regions. Ecological roles include habitat formation, nutrient cycling, and shoreline stabilization.

### Research-to-commercialization roadmap for tropical AOS

The successful transition of AOS derived from tropical seaweeds from laboratory research to commercial poultry feed applications requires a clearly defined and integrated development pathway. A structured roadmap is essential to ensure technical feasibility, economic viability, and sustainability.

#### Sustainable seaweed farming

The foundation of AOS commercialization lies in the sustainable cultivation of alginate-rich seaweeds, particularly genera such as *Sargassum* and *Kappaphycus* in tropical coastal regions. Seaweed farming initiatives should prioritize environmentally responsible practices, including site selection, controlled harvesting, and ecosystem preservation. Pilot programs involving local coastal communities are critical to promoting responsible cultivation, improving livelihoods, and ensuring a consistent supply of raw biomass.

#### Alginate extraction technologies

Efficient alginate extraction from harvested seaweed is a key step in the production chain. Innovation in extraction technologies should focus on maximizing yield while minimizing chemical inputs and environmental impact. Enzymatic hydrolysis using alginate lyases has emerged as a promising approach to enhance extraction efficiency and alginate quality [101]. Such techniques improve process sustainability and are particularly suitable for decentralized production systems in tropical regions.

#### Conversion of alginate to AOS

The conversion of alginate into bioactive AOS is primarily achieved through enzymatic depolymerization. The use of specific alginate lyases capable of selectively cleaving alginate polymers into low-degree-of-polymerization oligosaccharides can significantly enhance production efficiency and product consistency [102]. Optimizing enzyme selection and reaction conditions is essential to obtain AOS with desirable functional properties for poultry nutrition.

#### Encapsulated feed formulation

To ensure stability and bioavailability during feed processing and gastrointestinal transit, AOS should be incorporated into encapsulated feed formulations. Microencapsulation technologies protect AOS from thermal and mechanical degradation, facilitate controlled release, and enhance their prebiotic efficacy within the gut. Such formulations have been shown to improve nutrient utilization and gut health in poultry systems [103].

This roadmap highlights the importance of multidisciplinary collaboration among marine biotechnologists, agricultural engineers, feed technologists, and animal nutritionists to ensure successful commercialization while maximizing socio-economic benefits for local communities.

## EXTRACTION AND OLIGOSACCHARIDE PRODUCTION FOR FEED-GRADE USE

### Conventional alkaline and acid-based extraction methods

Alginate extraction from tropical brown seaweeds has gained increasing scientific attention due to its applications across food, pharmaceutical, and agricultural sectors. Conventional extraction methods include acid, alkaline, and calcium-based techniques. Among these, acid extraction has demonstrated high efficiency in *Turbinaria murayana*, yielding up to 26.93% alginate with favorable physicochemical properties, including moderate viscosity and molecular weight [104]. Variability in alginate yield is influenced by processing techniques; for instance, salt-reduced *T. murayana* contains approximately 13.51% alginate [105], while fermentation using fruit-based local microorganisms can increase alginate content to 40.93% [106].

Standard alkaline extraction typically involves acid pre-treatment, alkaline solubilization using NaOH or Na_2_CO_2_ solid–liquid separation, and precipitation [107, 108]. Although widely applied, this approach may lead to polysaccharide degradation and impurity contamination under high temperatures or prolonged processing [109, 110]. Hypochlorite treatment is commonly used to remove pigments and polyphenols [111].

### Advanced physical-assisted extraction techniques

Recent refinements include microwave-assisted extraction (MAE) and ultrasound-assisted extraction (UAE), which enhance alginate yield and purity while reducing processing time. MAE has been shown to improve alginate recovery from *Sargassum* species [112], whereas UAE accelerates extraction but may compromise polymer integrity if excessive energy is applied [113]. These techniques represent promising alternatives to conventional extraction when properly optimized.

### Enzyme-assisted and low-energy extraction approaches

Enzyme-assisted extraction offers a greener and more sustainable alternative to chemical-based methods. Enzymes such as cellulases, proteases, and alginate lyases facilitate cell wall degradation and reduce chemical consumption [114, 115]. This results in alginates with more favorable molecular characteristics and improved purity [116]. Alginate lyases from genera such as *Microbulbifer* and *Pseudoalteromonas* selectively cleave 1,4-glycosidic bonds, improving extraction efficiency while preserving molecular integrity [117–120]. These methods reduce chemical usage and maintain structural integrity [121, 122]. Cold-adapted alginate lyases function effectively at ambient temperatures, significantly lowering energy requirements [123, 124]. Combined enzymatic strategies and immobilized enzymes further enhance extraction efficiency and cost-effectiveness [125–127].

### Decentralized and small-scale extraction in tropical regions

Low-energy extraction methods such as MAE, UAE, and enzyme-assisted processes are particularly suitable for decentralized alginate production in tropical coastal communities [128]. Chelating agents like sodium citrate can replace harsh alkalis while maintaining extraction efficiency [129]. Smallholder feasibility has been demonstrated in studies involving local *Sargassum* and *Laminaria* species, optimized using response surface methodology [130, 131]. These approaches reduce costs, promote sustainability, and support local biotechnology development [110].

### Pilot-scale optimization and techno-economic considerations

At pilot-scale, alkaline extraction remains a critical step, with extraction parameters such as temperature, solvent concentration, and duration requiring careful optimization to balance yield and resource efficiency [132]. Advanced techniques such as reactive extrusion further enhance extraction performance by improving biomass–solvent interaction [88]. Environmental factors during seaweed growth, including light intensity, wave exposure, nutrient availability, and harvesting age, significantly influence postharvest alginate yield [133].

Economic feasibility remains a key consideration, with biomass cost and supply stability affecting large-scale production [134]. Techno-economic analyses indicate that integrated biorefinery frameworks can improve the financial viability of alginate extraction and AOS production [135].

### Relevance of extraction conditions to poultry nutrition outcomes

Extraction conditions strongly influence the molecular characteristics and bioactivity of feed additives. Studies on plant-derived bioactives, such as *Peronema canescens*, demonstrate that optimized extraction parameters yield compounds that enhance poultry growth performance, gut health, and immune regulation without adverse effects [136]. These molecular traits influence gene expression related to metabolism and disease resistance, highlighting the importance of integrating extraction technology with nutritional and genetic strategies to optimize poultry productivity [137, 138].

## DEPOLYMERIZATION TECHNIQUES FOR ALGINATE OLIGOSACCHARIDE PRODUCTION

### Enzymatic depolymerization using alginate lyases

The depolymerization of alginate into AOS is a critical step in enhancing its functionality and applicability in poultry feed. Among the available approaches, enzymatic depolymerization using alginate lyases is particularly effective due to its specificity and controllability. Enzymes belonging to the polysaccharide lyase family 7 (PL7) play a dominant role in AOS production and include both endo- and exo-type lyases, which determine the DP of the resulting oligosaccharides [124, 139].

Endo-type PL7 lyases cleave internal glycosidic bonds within alginate chains, producing AOS with a wide range of DP values, typically from disaccharides to decasaccharides. These oligosaccharides often contain unsaturated terminal residues that contribute to enhanced biological activity [140]. In contrast, exo-type lyases act on terminal residues and predominantly generate monosaccharides, as observed for enzymes such as VxAly7D [141, 142]. Bifunctional alginate lyases combine both endo- and exo-cleavage activities, allowing fine-tuning of DP and oligosaccharide profiles [139]. Mechanistically, alginate lyases operate through β-elimination reactions that introduce C4–C5 double bonds into uronic acid residues, thereby influencing enzyme specificity and DP distribution [143, 144].

### Physical depolymerization methods

Physical depolymerization techniques, including microwave irradiation and UAE, offer rapid processing and high extraction efficiency. Microwave treatment of *Sargassum* species, particularly when combined with mild acidic conditions, has been reported to increase alginate recovery by up to 36% [112]. UAE enhances cell wall disruption and mass transfer, improving extraction efficiency and reducing processing time [128]. However, these physical methods often result in heterogeneous molecular weight distributions and may compromise bioactivity due to excessive polymer degradation if not carefully controlled [145]. Consequently, while physical approaches are efficient, they lack the precision of enzymatic methods in producing AOS with predictable functional properties [146, 147].

### Sustainability advantages of enzymatic approaches

Compared with physical and chemical depolymerization, enzymatic methods are inherently more sustainable. They operate under mild reaction conditions, require minimal energy input, and significantly reduce the need for harsh chemicals [148]. The high substrate specificity of alginate lyases minimizes by-product formation and waste generation, aligning enzymatic depolymerization with green chemistry principles [127]. These advantages make enzyme-based AOS production particularly suitable for environmentally responsible feed-grade applications.

## INTEGRATED WORKFLOW FOR ALGINATE EXTRACTION AND AOS PRODUCTION

A generalized workflow for alginate extraction and enzymatic depolymerization from tropical seaweeds is outlined as follows:

1. Seaweed collection: Selection and harvesting of tropical brown seaweeds based on alginate content.
2. Pre-treatment: Washing of biomass to remove sand, salts, and surface impurities.
3. Extraction methods:
  (a) Alkaline extraction using NaOH to solubilize alginate from cell walls [132];
  (b) Acidic extraction to modify alginate solubility [149];
  (c) MAE to enhance yield and efficiency [112];
  (d) UAE to reduce solvent usage and processing time [109].
4. Purification: Filtration and precipitation, commonly using ethanol or calcium ions, to remove impurities [149].
5. Enzymatic depolymerization: Application of alginate lyases to convert sodium alginate into bioactive AOS [93].
6. Characterization: Physicochemical analyses to determine purity, molecular weight, and functional properties of AOS.

## SCALABILITY AND ENVIRONMENTAL CONSIDERATIONS

The scalability of alginate extraction and depolymerization from tropical seaweeds is influenced by extraction method selection, biomass availability, and infrastructure. Conventional alkaline extraction, while widely adopted, is energy-intensive and generates substantial chemical waste [132]. In contrast, microwave and ultrasound-assisted techniques offer more energy-efficient alternatives by significantly reducing processing time and resource consumption [109, 112]. However, large-scale implementation of these advanced technologies may require considerable capital investment and technical expertise.

Waste management remains a key challenge, particularly for alkaline extraction processes that produce large volumes of alkaline effluents. Sustainable strategies, such as valorizing extraction by-products as fertilizers or soil amendments, have been proposed to reduce environmental impact and improve overall process sustainability [149, 150].

## FORMULATION CHALLENGES AND FEED-COMPATIBILITY

### Stability issues during feed processing

Incorporation of AOS into poultry feed presents challenges related to thermal and mechanical stresses encountered during pelleting and extrusion. Elevated temperatures, shear forces, moisture, and pressure can induce hydrolysis or oxidation of AOS, leading to reduced bioactivity and shelf-life [15, 151, 152]. Interactions with other feed components may further affect AOS stability and bioavailability.

### Encapsulation and controlled delivery strategies

Encapsulation technologies, particularly microencapsulation using alginate matrixes, have been developed to preserve AOS integrity during feed processing and storage. Calcium-mediated crosslinking produces thermally stable microcapsules that protect AOS from degradation and enable controlled release in the GIT [153, 154]. Targeted delivery enhances gut health, immune modulation, and feed efficiency [155].

High encapsulation efficiencies have been reported for alginate-based systems. For example, Oninku *et al*. [156] documented encapsulation efficiencies of 97 ± 2.63% using alginate as a carrier, highlighting its effectiveness in stabilizing bioactive compounds [157].

### Advanced encapsulation technologies

Recent advances in spray-drying and hybrid biopolymer matrixes have further improved AOS delivery in poultry feeds. Crosslinked alginate systems combined with carrier agents such as maltodextrin and whey protein enhance microencapsulation efficiency and protect co-delivered bioactives, including probiotics, during gastrointestinal transit [158, 159]. Co-spray-drying produces stable microparticles that safeguard AOS against moisture and temperature fluctuations while enabling incorporation of synergistic additives such as enzymes and probiotics [153, 160].

Comprehensive summaries of alginate extraction, depolymerization, and feed-compatibility techniques are presented in Table 4 [15, 109, 110, 112, 114–117, 125, 126, 128, 129, 141, 142, 153, 160], while comparative data on extraction yield, energy consumption, and purity are provided in Table 5 [95, 109, 112, 132, 149, 161].

**Table 4:** Comparison of conventional and advanced extraction and depolymerization techniques for producing feed-grade alginate and alginate oligosaccharides.

Technique/Method	Description/Principle	Advantages	Limitations	Reference
Standard Alkaline Extraction	Use of NaOH/Na2CO3 to solubilize alginate from seaweed cell walls	Simple, cost-effective	Lower purity, degradation at high temps	[109, 110]
Optimized Microwave-Assisted Extraction	Use of microwave to accelerate solubilization and improve yield	Higher yield, faster processing	Requires equipment, risk of overprocessing	[114]
Ultrasound-Assisted Extraction	Sonication disrupts cell wall structures to release alginate	Improved extraction efficiency, short time	Potential structural degradation	[115]
Enzyme-Assisted Extraction (EAE)	Application of cellulase, protease, alginate lyases to release alginate	Eco-friendly, higher purity, reduced energy usage	Requires enzyme supply, longer reaction time	[116, 117]
Cold-Adapted Alginate Lyases	Enzymes functioning at low temperature to reduce energy input	Energy-efficient, suitable for tropical applications	Specific to substrate, costly purification	[125, 126]
PL7 Endo-Alginate Lyases	Cleaves internal bonds to form oligosaccharides (DP 2–10)	Tailored AOS production, improved bioactivity	Requires precise enzyme control	[141, 142]
PL7 Exo-Alginate Lyases	Cleaves terminal bonds to produce monosaccharides or short AOS	High specificity, suited for low DP AOS	May limit structural diversity	[141, 142]
Microwave Irradiation (Depolymerization)	Non-enzymatic thermal process to break alginate chains	High yield, fast process	Potential uncontrolled breakdown	[112]
Ultrasonic Depolymerization	Mechanical disruption and heating from ultrasonic waves	Quick release, enhanced extraction	Energy-intensive, inconsistent DP	[128]
Encapsulation (Ca-Alginate Beads)	Microencapsulation using Ca²^+^ crosslinking to protect AOS in feed	Thermal protection, targeted gut delivery	Adds processing steps	[153]
Co-Spray-Drying with Bioactives	Co-encapsulation of AOS and synergistic ingredients (e.g., probiotics)	Stabilizes AOS, enhances function	Needs optimization of drying parameters	[15, 160]
Chelating Agent Extraction (Na-Citrate)	Neutral-pH extraction using mild chelators to replace harsh alkali	Eco-friendly, suitable for coastal operations	Lower solubility at ambient conditions	[116, 129]

MAE = Microwave-assisted extraction, UAE = Ultrasound-assisted extraction, AOS = Alginate oligosaccharides. Enzymatic methods primarily employ alginate lyases. Sustainability assessment considers energy consumption, chemical usage, and waste generation.

**Table 5:** Extraction yield, energy requirements, and techno-economic considerations associated with different alginate production methods.

Extraction method	Yield	Energy consumption	Purity outcomes	Reference
Alkaline extraction	High (up to 90%)	High, requires significant heating and chemicals	Moderate to high	[132, 149]
Acidic extraction	Moderate (60-80%)	Moderate to high	Moderate	[149]
Microwave-assisted	High (often >90%)	Low, quick and efficient	High	[112]
Ultrasound-assisted	Moderate to high (depends on frequency)	Low, rapid extraction	High due to reduced solvent interaction	[109]
Enzymatic extraction	High, yields AOS effectively	Moderate, enzymatic reactions have lower energy needs	Very high since enzymes target specific bonds	[95, 161]

AOS = Alginate oligosaccharides. Energy consumption estimates are indicative and depend on scale and equipment. Costs are approximate and influenced by biomass availability, labor, and local infrastructure.

## LIFE-CYCLE ASSESSMENT (LCA) AND CARBON FOOTPRINT

Evaluation of the environmental impact of alginate and AOS production requires LCA approaches that account for energy use, greenhouse gas emissions, and water consumption. Tropical seaweeds such as *Sargassum* generally require lower energy inputs for cultivation due to favorable growth conditions, including warm waters and high solar irradiance [162]. In contrast, temperate seaweed species often necessitate additional energy for cultivation and processing, resulting in a higher carbon footprint per unit of alginate produced [163].

Studies indicate that tropical alginate production, when combined with environmentally friendly extraction methods and renewable energy sources, can achieve lower overall greenhouse gas emissions [163, 164]. Nevertheless, direct comparative LCAs between tropical and temperate alginate production systems remain limited and warrant further investigation.

## CHEMISTRY AND PHYSICOCHEMICAL PROPERTIES RELEVANT TO THE POULTRY DIGESTIVE TRACT

### Structural determinants of AOS functionality

AOS possess distinct structural characteristics, including DP, mannuronic-to-guluronic acid (M/G) ratio, and limited branching, which collectively govern their physicochemical behavior. These parameters critically influence solubility, viscosity, gelation potential, and bioavailability within the poultry GIT, thereby determining their functional efficacy as feed additives.

Oligosaccharides with a low DP (typically <5) exhibit enhanced intestinal accessibility and bioavailability, facilitating efficient fermentation by beneficial gut microbiota [165]. In poultry, this promotes increased production of short-chain fatty acids (SCFAs), which support intestinal health. In contrast, AOS with higher DP values are less likely to be absorbed intact and are preferentially fermented in distal intestinal segments rather than the small intestine [166].

## MOLECULAR WEIGHT, SOLUBILITY, AND VISCOSITY

The molecular architecture of AOS plays a decisive role in their performance in both feed matrixes and the intestinal environment. DP strongly influences viscosity and solubility, with high-DP AOS exhibiting increased viscosity due to greater chain entanglement [15, 167]. Conversely, AOS with DP values ranging from 2 to 10 display lower viscosity and higher aqueous solubility, properties that are advantageous for poultry feed applications [15].

**Figure 2:**
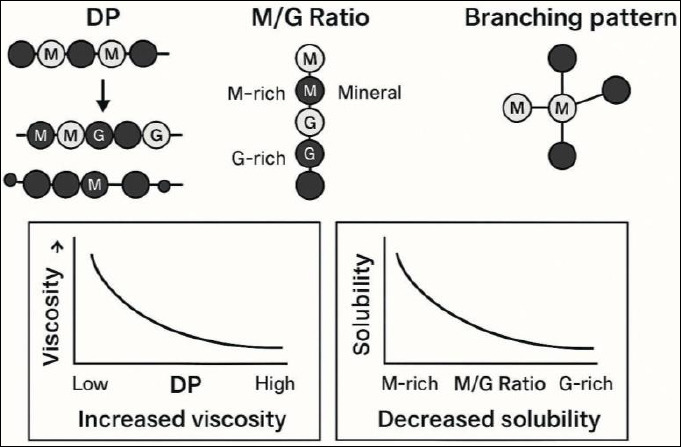
Schematic representation of key structural features of alginate, including degree of polymerization (DP), mannuronic/guluronic acid (M/G) ratio, and branching pattern, and their influence on physicochemical properties. Higher DP is associated with increased viscosity, whereas a shift from M-rich to G-rich alginate is linked to decreased solubility.

The M/G ratio further modulates these characteristics. High guluronic acid content promotes gel formation through ionic crosslinking with divalent cations, whereas increased mannuronic acid content enhances solubility and molecular flexibility while reducing viscosity [119, 168–170]. This balance enables the fine-tuning of AOS functionality for optimal feed performance. As illustrated in Figure 2, AOS consist of mannuronate (M) and guluronate (G) units arranged in shorter chains than native alginate polymers, accounting for their higher water solubility and distinct physicochemical behavior.

Although alginate polymers are primarily linear, limited branching can influence solvation capacity and viscosity. Branched AOS may exhibit slightly higher viscosity and confer additional biological functions, including modulation of gut microbial communities [15, 165]. In feed formulation, moderate viscosity improves feed texture, pellet integrity, and moisture retention, enhancing palatability and nutrient uptake [15, 171–174]. Within the intestine, viscous AOS can coat the mucosal surface, promoting nutrient absorption, microbial balance, and immune protection [17, 171, 175].

## SPECTROSCOPIC CHARACTERIZATION AND STRUCTURE–FUNCTION RELATIONSHIPS

Fourier-transform infrared (FTIR) spectroscopy is a key analytical tool for identifying functional groups in AOS that contribute to biological activity and microbial interactions [176, 177]. FTIR analyses have revealed structural variations among alginates derived from different seaweed sources and extraction methods [78]. Nuclear magnetic resonance spectroscopy further elucidates polysaccharide backbone configurations and their correlation with prebiotic efficacy [29]. These techniques also provide insight into interactions between AOS and other feed components, which may enhance nutrient absorption and gut health [178].

Research indicates that appropriate viscosity levels of AOS facilitate nutrient availability while limiting pathogen colonization in the poultry GIT [179]. Quantitative measurements of viscosity under different physiological conditions would further strengthen understanding of the mechanisms underlying AOS-mediated gut health benefits [99, 176].

### Physiological functions in poultry

The physicochemical properties of AOS translate into several key physiological effects in poultry:

1. Prebiotic activity: AOS selectively stimulate beneficial gut microbiota, serving as fermentable substrates that promote SCFA production, thereby enhancing gut integrity, nutrient absorption, and immune function [33, 180].
2. Immune modulation: Dietary AOS have been shown to upregulate immunoglobulin expression and antimicrobial peptide production, strengthening gut barrier function and defense against pathogens such as *Salmonella enterica* [180].
3. Viscosity-mediated gut function: Increased intestinal viscosity can slow digesta transit, improving nutrient absorption and favoring colonization by beneficial microbial populations over pathogenic species [15, 181].

From an economic perspective, local production of AOS offers a cost-effective alternative to imported AGPs. Incorporation of seaweed-derived AOS has been associated with improved poultry performance and egg quality, translating into tangible economic benefits for producers [182]. Furthermore, localized seaweed farming can reduce dependence on volatile global feed supply chains [178].

### Behavior across the pH gradient of the poultry GIT

The poultry GIT presents a dynamic pH gradient, ranging from near-neutral conditions in the crop (pH 6–7), highly acidic environments in the proventriculus and gizzard (pH 2–4), and a return to neutral-pH in the intestine (pH 6–7). AOS must retain structural integrity across these conditions to exert their functional effects.

In the crop, AOS remain stable, preserving viscosity and bioactivity [183]. Although acidic conditions in the proventriculus and gizzard pose a risk of degradation, hydrogen bonding within AOS structures confers partial resistance to acid hydrolysis, particularly when encapsulated [184]. Upon entry into the intestine, neutral-pH conditions restore solubility, promoting swelling and release of bioactive components [185, 186]. These transitions enhance targeted delivery and support the proliferation of beneficial bacteria such as *Lactobacillus* and *Bifidobacterium*, while suppressing pathogenic populations and optimizing nutrient absorption [183, 185, 186].

### Ion interactions and gelation potential

Guluronic acid-rich regions within alginate exhibit a strong affinity for divalent cations, particularly Ca²^+^ and Mg²+ forming gel networks through ionic crosslinking described by the “egg-box” model [15]. Calcium ions produce stronger and more stable gels than magnesium due to superior coordination with G-blocks [103]. Calcium alginate gels can destabilize under neutral intestinal pH conditions, enhancing solubility and bioavailability of co-delivered bioactive compounds [187].

Microbial fermentation of AOS in the intestine generates SCFAs such as butyrate, which strengthen gut integrity, modulate immune responses, and improve energy utilization [16, 188]. Gelation also supports mucosal protection by facilitating sustained nutrient release and enhancing bioadhesion to the intestinal lining [189, 190]. Collectively, these ion-mediated properties enable controlled delivery, immune modulation, and microbial homeostasis, positioning AOS as effective and sustainable alternatives to AGPs in poultry production systems [191–193].

## METABOLISM OF ALGINATE AND AOS IN POULTRY

### Gastrointestinal passage and resistance to digestion

**Figure 3:**
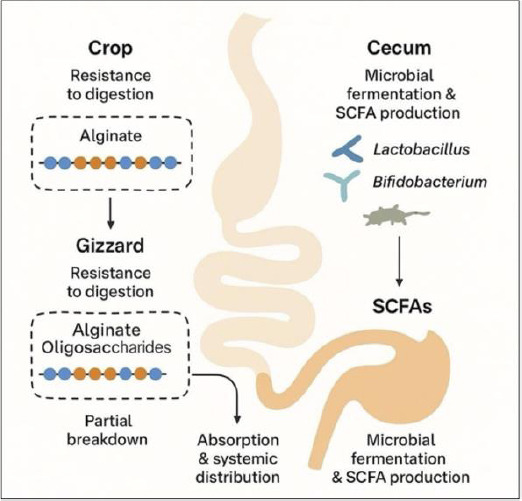
Proposed mechanism of alginate digestion, fermentation, and metabolic fate in the avian gastrointestinal tract. Alginate resists enzymatic digestion in the crop and gizzard, undergoes partial breakdown into alginate oligosaccharides, and is subsequently fermented by cecal microbiota, including *Lactobacillus* and *Bifidobacterium*, leading to short-chain fatty acid production, with partial absorption and systemic distribution.

A schematic representation of alginate metabolism can be seen in Figure 3. Alginate and its depolymerized derivatives, AOS, exhibit a high degree of resistance to enzymatic digestion throughout the poultry GIT, a property that underpins their role as functional prebiotics. This resistance is primarily attributed to the absence of endogenous alginate-degrading enzymes in poultry, particularly within the crop, gizzard, and small intestine [194]. Consequently, alginate polymers and AOS largely retain their structural integrity during early digestive transit.

Within the crop (pH 5–7) and the highly acidic environment of the gizzard (pH 2–4), alginates are subjected to mechanical agitation and chemical stress. Nevertheless, higher-molecular-weight alginates and structurally stable AOS are largely resistant to acid hydrolysis under these conditions [195, 196]. As digesta progresses into the small intestine, where pH conditions are closer to neutral, partial degradation may occur through microbial activity, particularly for shorter-chain AOS with favorable mannuronic-to-guluronic acid (M/G) ratios that enhance microbial accessibility [125, 197].

In addition, G-block-rich alginates interact with divalent cations such as Ca²^+^ and Mg²^+^, promoting gelation and bioadhesion to the intestinal epithelium. This interaction prolongs residence time within the gut, enabling gradual release of bioactive components and facilitating improved nutrient absorption and immune modulation [190–192]. Collectively, the digestive resistance and controlled intestinal persistence of AOS support their functional efficacy as prebiotic feed additives in poultry.

## MICROBIAL FERMENTATION AND SHORT-CHAIN FATTY ACID PRODUCTION

Upon reaching the ceca, AOS undergo fermentation by commensal gut microbiota, notably Lactobacillus and Bacteroides species, which possess alginate lyases capable of degrading AOS into absorbable monosaccharides and metabolic intermediates [198, 199]. This microbial fermentation leads to the production of SCFAs, primarily acetate, propionate, and butyrate, which play central roles in maintaining intestinal health.

The butyryl-CoA:acetate CoA-transferase pathway is particularly important in acetate and butyrate biosynthesis. Butyrate serves as the primary energy source for colonocytes and reinforces gut barrier integrity, while acetate and propionate contribute to systemic energy metabolism and immune regulation [200–202]. Increased butyrate concentrations have been associated with enhanced microbial diversity and elevated populations of *Lactobacillus*, reflecting a positive feedback loop between SCFA production and microbiota modulation [203–205].

Beyond SCFA generation, AOS fermentation promotes beneficial shifts in microbial composition. Dietary AOS supplementation consistently enriches populations of *Lactobacillus* and *Bifidobacterium*, which suppress pathogenic bacteria through competitive exclusion, acidification, and bacteriocin production [205–207]. These microbiota-driven effects strengthen gut integrity, improve nutrient utilization, and enhance immune responsiveness, collectively contributing to improved feed efficiency and growth performance in poultry [208, 209].

## COMPARISON WITH ESTABLISHED PREBIOTIC COMPOUNDS

The prebiotic effects of AOS share functional similarities with established oligosaccharides such as mannan oligosaccharides (MOS), fructooligosaccharides (FOS), galactooligosaccharides (GOS), and β-glucans. MOS have been widely reported to stimulate beneficial gut microbiota while inhibiting pathogen adhesion, thereby supporting gut health in poultry [210]. GOS modulate intestinal microbial communities and immune responses, promoting beneficial bacterial populations in chicks [211]. FOS have been associated with improved body weight gain and feed conversion efficiency in broilers, partly through inhibition of pathogen adhesion, including *Salmonella* and *Campylobacter*, to intestinal epithelial cells [212]. β-glucans, meanwhile, contribute to oxidative stress mitigation and immune enhancement, particularly under environmental or physiological stress [213].

While these prebiotics are well established, AOS offer distinct advantages due to their unique structural features, fermentation profiles, and multifunctional effects, positioning them as promising alternatives or complements within antibiotic-free poultry feeding strategies.

## ABSORPTION, SYSTEMIC DISTRIBUTION, AND EXCRETION

Recent evidence suggests that low-molecular-weight AOS, typically composed of 2–25 monomer units, may be absorbed in the small intestine due to their high solubility and favorable physicochemical properties [214, 215]. Structural features such as the M/G ratio influence epithelial uptake, with guluronic acid-rich AOS exhibiting enhanced cellular interaction and bioavailability [215, 216].

However, it is important to note that much of the current understanding of AOS absorption is extrapolated from mammalian models rather than derived directly from poultry studies. Empirical data on absorption kinetics and systemic availability of AOS in avian species remain limited, underscoring the need for poultry-specific investigations [217].

Following absorption, AOS can enter systemic circulation and reach peripheral tissues, including the liver and muscle, where they may influence metabolic and immune pathways [218, 219]. Systemically, AOS have been shown to activate macrophages, modulate inflammatory cytokine expression, and enhance antioxidant defenses via TLR signaling pathways, particularly TLR4 [17, 220]. Activation of TLR4 triggers downstream NF-κB and MAPK signaling cascades, which regulate the expression of immune and inflammatory mediators. Crosstalk between TLR4 and other TLRs further fine-tunes immune responses, shaping the overall immunological landscape [221].

Precise absorption and excretion rates in poultry remain poorly quantified and warrant further targeted research [222]. Despite partial absorption, a substantial proportion of AOS remains unabsorbed and is either fermented by gut microbiota or excreted in feces. While small amounts of conjugated uronic acid metabolites may be detected in urine following hepatic processing, fecal excretion constitutes the dominant elimination route [16, 223].

Optimizing AOS structural parameters, such as DP and M/G ratio, along with encapsulation and formulation strategies, may enhance bioavailability, reduce losses, and maximize functional outcomes [17, 224]. Balancing absorption, microbial utilization, and excretion is therefore critical for designing effective AOS-based feed additives in poultry nutrition.

## TRANSLATIONAL POTENTIAL OF TROPICAL AOS

### Applications beyond poultry: Aquaculture and livestock systems

AOS have demonstrated considerable translational potential across multiple animal production systems. In aquaculture, AOS function as effective prebiotic agents that positively modulate gut microbiota and enhance overall health in aquatic species. Studies have reported improvements in growth performance, feed efficiency, and immune responses in marine shrimp, particularly *Penaeus vannamei*, following dietary supplementation with algae-derived AOS [225]. Similarly, favorable alterations in intestinal microbial composition and enhanced growth have been observed in *Fenneropenaeus indicus* [226].

Beyond aquaculture, AOS applications extend to terrestrial livestock species, including swine and ruminants. In pigs, microbial fermentation of AOS leads to increased production of SCFAs, which play key roles in maintaining gastrointestinal integrity and metabolic health [227]. Moreover, AOS supplementation has been associated with modulation of systemic immune responses in livestock, highlighting their dual role in promoting gut health and improving overall productivity [68].

### Interdisciplinary research and innovation pathways

Realizing the full potential of tropical seaweed-derived AOS requires integrated, interdisciplinary research approaches. Collaboration between marine biotechnology, animal nutrition, and environmental engineering can facilitate innovative and sustainable applications of AOS in animal agriculture. Marine biotechnology offers tools to optimize extraction, depolymerization, and scale-up processes, ensuring consistent production of high-quality AOS from tropical seaweeds [15]. Concurrently, advances in animal nutrition can guide the formulation of species-specific AOS supplements designed to target defined gut health and performance outcomes, improving feed palatability and digestibility [228]. Environmental engineering perspectives are essential for evaluating ecosystem impacts, resource efficiency, and life-cycle sustainability, ensuring that AOS integration aligns with environ-mentally responsible farming practices.

## FUNCTIONAL EFFECTS OF AOS ON POULTRY PERFORMANCE

### Growth performance and feed efficiency

AOS have shown substantial functional benefits in poultry nutrition, particularly in improving ADG, FCR, and overall nutrient utilization. Acting as prebiotics, AOS selectively promote beneficial gut microbiota such as *Lactobacillus* and *Bifidobacterium*, thereby enhancing gut health, nutrient absorption, and growth performance [229].

Several studies report improvements in FCR following AOS supplementation, reflecting enhanced feed efficiency. However, reported magnitudes of weight gain vary considerably among studies, and consistent increases of 5%–10% compared with controls are not uniformly supported in the literature. Instead, growth responses appear dependent on diet composition, AOS structure, and inclusion level [230, 231]. Meta-analytical assessments suggest that AOS contribute to improved ADG and FCR, although the extent of improvement differs across experimental conditions [232].

### Nutrient digestibility and antioxidant effects

AOS supplementation enhances nutrient digestibility by modulating gut microbial activity and supporting intestinal function. Improved protein digestibility is associated with microbial enzyme production, while enhanced energy and fat utilization are linked to SCFA-mediated improvements in gut morphology and lipid metabolism [23, 176]. Mineral absorption, particularly of calcium and magnesium, is also improved, as AOS gel-forming properties facilitate ion solubilization and transport within the intestine [23, 176].

In addition to digestive benefits, AOS exhibit antioxidant properties that mitigate oxidative stress and inflammation in poultry. Increased activity of antioxidant enzymes such as superoxide dismutase (SOD), catalase, and glutathione peroxidase (GSH-Px) has been reported in broilers receiving AOS-supplemented diets, although variability exists across studies [231]. These effects contribute to improved metabolic health and resilience under production stress.

### Mechanistic basis for growth promotion

Broilers fed AOS-supplemented diets often show statistically significant increases in ADG relative to control groups. These effects are attributed to improved nutrient absorption, reduced pathogenic load, and enhanced epithelial integrity supported by SCFAs generated during microbial fermentation [23, 176]. AOS have been shown to play a role in modulating gut health, enhancing microbiota balance, and exhibiting prebiotic activity, which collectively contribute to improved nutrient digestibility and utilization [231]. Improved FCR reflects reduced feed intake per unit of weight gain, as sustained SCFA production lowers digestive energy demands and redirects energy toward growth [234].

AOS also positively influence blood biochemical parameters and cecal microbial populations, further supporting nutrient bioavailability and gut health. Osman *et al*. [235] demonstrated that prebiotic supplementation improved metabolic indices and microbial balance, contributing to enhanced growth performance in broiler chickens.

### Dose-dependent responses and optimal inclusion levels

The effects of AOS on poultry performance are strongly dose-dependent [236]. Higher inclusion levels have generally been associated with greater improvements in gut health and immune function [237]. Continuous supplementation may enhance nutrient digestibility and microbiota stability, contributing to sustained improvements in feed efficiency [238]. However, prolonged use without dietary rotation may result in diminishing returns, underscoring the importance of optimized dosing strategies [239].

Threshold levels refer to the minimum effective concentration required to elicit beneficial biological responses without adverse effects [240]. Several studies suggest that inclusion levels around 1.0 g/kg diet effectively enhance immune parameters and growth performance, providing a practical benchmark for poultry diets [240]. Reported responses vary with dosage: supplementation at 0.05% has been associated with improvements in ADG and FCR; 0.1% enhances immune markers and modestly increases egg production; 0.2% produces more pronounced gains in ADG and FCR; and inclusion levels between 0.5% and 1.0% yield the most consistent improvements in growth and productivity, particularly under environmental or management stress [241].

### Limitations and future research needs

Despite substantial evidence supporting the growth-promoting effects of AOS, several limitations remain. Long-term studies under commercial production conditions are limited, and few investigations have evaluated stage-specific responses across different growth phases [236]. Variability in diet composition, gut microbiota, and environmental factors also contributes to inconsistent findings regarding optimal inclusion levels and performance outcomes [217]. Future research should focus on long-term field trials, dose optimization, and interactions with other functional feed additives to fully elucidate the role of AOS in sustainable poultry production.

## EFFECTS OF AOS ON EGG PRODUCTION AND QUALITY

### Egg production performance and shell quality

In laying hens, dietary supplementation with AOS has been shown to improve laying rate, egg mass, and overall egg quality. These benefits are largely attributed to enhanced intestinal morphology, including increased villus height and a more favorable gut microbial balance, which together improve nutrient absorption and utilization [242]. Such effects are particularly important during later stages of the laying cycle, when productivity and shell quality typically decline.

Improvements in eggshell quality are consistently reported following AOS supplementation, with increased shell thickness and breaking strength. Enhanced bioavailability and intestinal absorption of minerals, particularly calcium, support more efficient shell mineralization and structural integrity [243, 244]. These attributes contribute directly to reduced egg breakage and improved economic returns for producers.

### Yolk coloration and pigment deposition

AOS supplementation has also been associated with enhanced yolk color, an important quality attribute that influences consumer preference. AOS facilitates the intestinal absorption and stabilization of carotenoids such as lutein and zeaxanthin derived from carotenoid-rich feed ingredients. Improved carotenoid uptake results in deeper yellow yolk pigmentation [245–247].

Furthermore, AOS protects carotenoids from oxidative degradation during digestion and storage, allowing greater deposition into the yolk [248, 249]. This antioxidant-mediated stabilization not only improves yolk color but may also enhance the nutritional value of eggs.

## IMMUNE RESPONSE MODULATION IN POULTRY

AOS exert immunomodulatory effects by enhancing both systemic and mucosal immune responses. Increased expression of cytokines such as interleukin-6 (IL-6) promotes immune cell activation, while elevated secretory immunoglobulin A (sIgA) strengthens intestinal barrier function [250–252].

The enhanced sIgA response is particularly important for preventing pathogen adhesion and epithelial colonization, providing protection against enteric diseases such as coccidiosis and salmonellosis. Several studies report reduced pathogen load and lower disease incidence in poultry receiving AOS-supplemented diets [253–256].

Mechanistically, AOS promotes the proliferation of beneficial commensal bacteria that outcompete pathogenic microorganisms and stimulate SCFA production. These metabolites reinforce gut barrier integrity and activate TLR-mediated innate immune signaling pathways, amplifying host immune defenses [257, 258].

## GUT HEALTH AND INTESTINAL MORPHOLOGICAL ADAPTATIONS

AOS supplementation induces significant improvements in intestinal morphology, including increased villus height, deeper crypts, and elevated goblet cell numbers. Taller villi expand the absorptive surface area, while deeper crypts reflect enhanced epithelial cell renewal capacity [259–261].

Goblet cells, which produce protective mucus, are also increased following AOS supplementation. A thicker mucus layer provides a physical barrier against pathogen invasion and mechanical damage [223, 262]. In addition, AOS upregulates tight junction proteins such as zonula occludens-1 (ZO-1) and occludin, improving epithelial integrity and selective permeability [261, 263]. These changes reduce pathogen translocation and support gut homeostasis.

AOS also exhibits anti-inflammatory effects by reducing the expression of pro-inflammatory cytokines, including tumor necrosis factor-α (TNF-α) and IL-6. This anti-inflammatory action complements the prebiotic function of AOS in maintaining a balanced and resilient gut microbiome [223].

## EVIDENCE OF EFFICACY AND PRODUCTION-LEVEL IMPACT

Substantial evidence supports the efficacy of AOS as a functional feed additive influencing growth performance, FCR, gut health, and egg quality. Sittiya *et al*. [264] demonstrated significant improvements in growth performance and gut development in broiler chickens supplemented with AOS. Similar findings indicate that crude oligosaccharides modulate gut microbiota, enhancing nutrient absorption and immune function [265].

Effective dietary inclusion levels of AOS generally range from 1%–2% of the total diet, resulting in favorable effects on growth performance and nutritional efficiency [264]. Importantly, excessive inclusion levels may diminish benefits, highlighting the need for dosage optimization. Oligosaccharides derived from natural sources, including bamboo, have also demonstrated strong prebiotic activity without adverse effects on growth performance [265].

Comparative studies suggest that AOS can effectively substitute AGPs and complement or replace probiotics and other prebiotics. While probiotics such as *Bacillus amyloliquefaciens* can improve gut health, their efficacy may vary under commercial conditions [266]. In contrast, AOS often produce more consistent improvements in FCR and body weight gain by selectively enhancing beneficial microbiota [267]. Given concerns regarding antimicrobial resistance and residue accumulation associated with AGPs, AOS represent a sustainable and consumer-acceptable alternative [268].

## ANTIOXIDANT STATUS AND RESISTANCE TO OXIDATIVE STRESS

AOS have demonstrated the capacity to modulate oxidative stress biomarkers in poultry, contributing to improved health and productivity. Supplementation with AOS has been associated with increased activity of antioxidant enzymes such as SOD and GSH-Px, alongside reduced malondialdehyde (MDA) concentrations, a marker of lipid peroxidation [231].

These antioxidant effects enhance redox balance and protect cellular integrity, particularly under stress conditions such as heat exposure or disease challenge. AOS has been shown to activate nuclear factor erythroid 2–related factor 2 (Nrf2) signaling pathways, promoting the expression of antioxidant genes and improving meat quality in heat-stressed broilers [269, 270].

Infectious challenges, including coccidiosis, induce substantial oxidative stress and inflammation in poultry [271]. Dietary supplementation with prebiotic-rich AOS enhances immune competence and supports gut microbial stability, reducing susceptibility to infection and disease-associated oxidative damage [272]. This dual antioxidant and immunomodulatory functionality reduces reliance on AGPs and aligns with sustainable poultry production practices [272].

Collectively, in vivo poultry studies summarized in Table 6 [23, 176, 188, 229, 242, 245, 246, 250, 252, 254, 255, 260, 262, 273, 274] demonstrate that AOS supplementation consistently improves growth performance, nutrient digestibility, egg production, gut morphology, antioxidant status, and immune responses, while reducing

**Table 6:** Summary of *in vivo* studies evaluating the effects of alginate oligosaccharide supplementation on growth performance, gut health, immune response, and antioxidant status in poultry.

Study focus	Poultry type	Key findings	AOS Source/Dose	Reference
Growth performance (ADG, FCR)	Broilers	Significant improvement in ADG and FCR	AOS, 0.1–0.5% in feed	[229]
Nutrient digestibility (protein, energy, fat)	Broilers	Improved digestibility of macronutrients and mineral absorption	AOS from brown algae	[23, 176]
Egg production	Layers	Increased laying rate, egg weight, and yolk color	AOS, 0.3 mg/kg selenium in ration	[242]
Yolk pigmentation and carotenoid absorption	Layers	Enhanced yolk color and carotenoid uptake	AOS with carotenoid-rich diet	[245, 246, 273]
Immune modulation (IL-6, sIgA)	Broilers and layers	Upregulation of IL-6 and sIgA, improved immune resilience	AOS 0.5% oral	[250, 252]
Protection against enteric pathogens	Broilers	Reduced Salmonella and Eimeria colonization	AOS from seaweed	[254, 255]
Gut morphology (villus height, goblet cells)	Broilers	Increased villus height and crypt depth; enhanced mucus layer	AOS, dietary inclusion	[188, 260, 262]
Antioxidant status	Broilers and layers	Modulation of SOD, GSH-Px, MDA levels	Tropical AOS	[188, 274]

AOS = Alginate oligosaccharides, ADG = Average daily gain, FCR = Feed conversion ratio, GSH-Px = glutathione peroxidase, IL-6 = Interleukin-6, MDA = Malondialdehyde, sIgA = Secretory immunoglobulin A, SOD = superoxide dismutase, SCFA = Short-chain fatty acids. Effects are compared with unsupplemented control diets unless otherwise stated.

## AOS AS ALTERNATIVES TO AGPS

### Mechanistic parallels between AOS and AGPs

**Figure 4:**
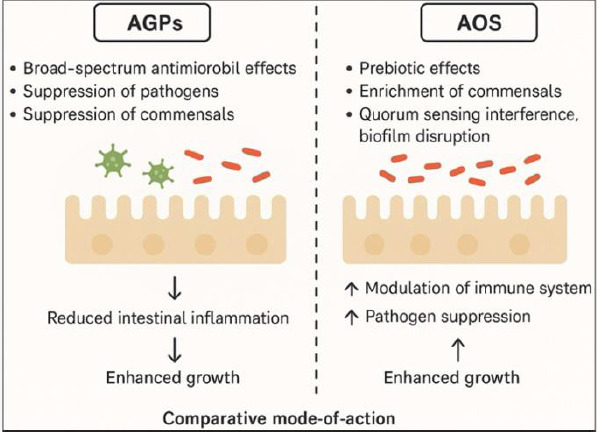
Comparative modes of action of algal-derived growth promoters (AGPs) and alginate oligosaccharides (AOS) in the intestinal environment. AGPs exert broad-spectrum antimicrobial activity that suppresses both pathogenic and commensal microorganisms, leading to reduced intestinal inflammation and enhanced growth. In contrast, AOS primarily act through prebiotic mechanisms by selectively enriching beneficial microbiota, interfering with quorum sensing and biofilm formation, modulating host immune responses, and suppressing pathogens, ultimately promoting improved growth performance.

AOS have emerged as promising alternatives to AGPs in poultry production due to their multifactorial modes of action that enhance growth performance, immune competence, and gut health. Similar to AGPs, AOS modulate intestinal microbiota, suppress pathogenic bacteria, and support host immunity through both direct and indirect mechanisms. Figure 4 illustrates a comparative overview of the mechanisms of action of AOS relative to other bio-based feed additives, highlighting their influence on gut health, immune modulation, antioxidant activity, and nutrient absorption.

A primary mechanism of AOS is their prebiotic effect, whereby they selectively stimulate the growth of beneficial gut microorganisms such as *Lactobacillus* and *Bifidobacterium* [275, 276]. This selective enrichment competitively excludes pathogenic bacteria and enhances the production of SCFAs, creating an intestinal environment unfavorable to pathogens such as *Salmonella* spp. and *Eimeria* spp. [218].

In terms of immune modulation, AOS has been shown to stimulate cytokine production, including IL-6, which enhances macrophage activation and T-cell-mediated immune responses [277, 146]. Concurrently, AOS regulates inflammatory processes by downregulating excessive pro-inflammatory cytokine expression, contributing to immune homeostasis [218]. Enhanced mucosal immunity is further supported by increased secretion of immunoglobulin A (sIgA), strengthening the intestinal barrier against pathogen invasion [99, 278].

### Intestinal barrier protection and antimicrobial actions

AOS contributes to intestinal barrier integrity by promoting goblet cell proliferation and reinforcing tight junction protein expression, thereby improving mucus secretion and epithelial resilience [256, 261]. These structural improvements reduce pathogen translocation and systemic infection risk [158].

Beyond indirect microbial modulation, AOS also exhibits direct antimicrobial properties. Notably, AOS interferes with quorum-sensing (QS) signaling pathways by inhibiting acyl homoserine lactones, thereby disrupting bacterial communication and biofilm formation [279, 280]. Since biofilms often confer resistance to antibiotics, their disruption by AOS enhances microbial susceptibility and reduces persistence within the gut ecosystem [281]. Collectively, these mechanisms closely parallel those of AGPs, positioning AOS as functionally comparable feed additives.

## COMPARATIVE EFFICACY OF AOS AND CONVENTIONAL AGPS

### Growth performance and feed efficiency

Several *in vivo* studies have directly compared the efficacy of AOS with commonly used AGPs such as bacitracin and virginiamycin. In broiler chickens, AOS supplementation has been associated with significant improvements in ADG and FCR. Chen *et al*. [282] reported increased ADG in broilers receiving AOS, while Qiu *et al*. [283] observed FCR improvements comparable to those achieved with virginiamycin supplementation.

Similar to organic acids and essential oils, other recognized AGP alternatives, AOS supports digestive health and growth performance during both starter and finisher phases [284]. These performance gains are attributed to enhanced nutrient absorption and improved microbial balance in the GIT, mediated by the prebiotic effects of AOS [285].

### Physiological and antioxidant effects

Mechanistically, AOS supplementation promotes increased villus height and crypt depth, expanding the absorptive surface area of the intestine and improving nutrient uptake efficiency [286, 287]. In addition, AOS reduces oxidative stress and intestinal inflammation by modulating antioxidant and oxidative stress markers such as SOD, glutathione peroxidase (GSH-Px), and MDA [288]. These physiological effects mirror those induced by AGPs but without contributing to antimicrobial resistance.

### Limitations in comparative evaluation

Despite encouraging findings, direct comparisons between AOS and AGPs across studies remain challenging due to methodological variability. Differences in experimental design—including bird age, dietary composition, housing systems, and environmental conditions—substantially influence outcomes [289, 290]. Moreover, inconsistencies in defining and measuring performance indicators such as ADG and FCR complicate cross-study comparisons and meta-analytical assessments [291].

Variations in AOS and AGP dosages, formulations, and feed matrixes further confound interpretations [292]. Importantly, fundamental mechanistic differences exist between AOS and AGPs: AOS primarily function through targeted microbiota modulation and immune enhancement, whereas AGPs exert broad-spectrum antimicrobial effects [293]. These distinctions can lead to different physiological and ecological outcomes within the gut.

### Regulatory context and sustainability considerations

Increasing global regulatory scrutiny over AGP use, coupled with rising concerns regarding antimicrobial resistance, has accelerated the transition toward antibiotic-free poultry production systems [294]. In this context, AOS offers a safe and effective alternative capable of improving growth performance and health without promoting resistance development [295, 296].

Comparative performance data summarized in Table 7 [289, 297–303] demonstrate that AOS performs comparably to, or better than, common AGPs such as bacitracin, virginiamycin, and zinc bacitracin in terms of FCR, ADG, and immune modulation. Notably, AOS enhances immune responses while preserving beneficial gut microbiota, an advantage not consistently observed with AGPs.

**Table 7:** Comparative effects of AOS and conventional antibiotic growth promoters on poultry performance and health indicators.

Additive	FCR improvement	ADG improvement	Immune markers	Reference
AOS	Increased efficiency noted	Enhanced body weight gain reported	Modulation of immune response observed	[297–299]
Bacitracin	Significant improvements	Notably increased weight gain	Downregulation of pro-inflammatory cyotkines	[289, 300, 301]
Virginiamycin	Effective for FCR management	Consistent ADG increases noted	Immunomodulatory effects noted	[289, 302]
Zinc bacitracin	Positive impact on FCR	Facilitates body mass improvements	Adjusments in gut microbiota beneficial	[290, 302, 303]

AOS = Alginate oligosaccharides, AGP = Antibiotic growth promoter, FCR = Feed conversion ratio, ADG = Average daily gain. Performance outcomes are influenced by bird age, diet composition, inclusion level, and management conditions.

### Comparison with specific AGPs

Bacitracin primarily targets gram-positive bacteria by inhibiting peptidoglycan synthesis and compromising bacterial cell wall integrity [297]. While effective in enhancing growth performance and nutrient absorption, bacitracin use has been associated with shifts in microbial resistance profiles [304]. Virginiamycin operates through a different mechanism, disrupting bacterial protein synthesis via ribosomal interference [305]. However, similar to bacitracin, its use has been linked to increased antimicrobial resistance in gut microbial populations [306].

In contrast, AOS provides broader biological benefits, including antioxidant activity, gut barrier enhancement, and immune modulation, without exerting selective pressure that promotes resistance [307, 308]. As a renewable, marine-derived biopolymer, AOS also aligns with sustainability goals and the global shift toward environmentally responsible feed additives [309, 310]. Nevertheless, challenges remain for AOS and other bio-based adhesives, particularly in achieving mechanical properties comparable to synthetics and maintaining performance under variable environmental conditions, while lignin-based systems continue to struggle with water resistance and formulation consistency [311].

## FUTURE DIRECTIONS AND RESEARCH NEEDS

Future research should prioritize long-term, standardized trials directly comparing AOS and AGPs under controlled and commercial production conditions. Such studies are essential to accurately assess sustained effects on growth performance, immunity, gut health, and mortality [312]. Comprehensive meta-analyses and controlled-environment experiments will further help resolve inconsistencies and strengthen the evidence base supporting AOS as reliable AGP replacements [313, 314].

Performance comparisons summarized in Table 8 [33, 243, 248, 280–284, 315–318] emphasize that AOS supplementation can match or surpass AGPs across multiple parameters, including ADG, FCR, yolk pigmentation, microbiota balance, immune response, and mortality reduction. A unique advantage of AOS is its ability to inhibit QS and biofilm formation, mechanisms not provided by conventional AGPs, underscoring its potential as a next-generation functional feed additive.

**Table 8:** Comparative effects of AOS and conventional AGP on growth performance, gut health, immune response, antioxidant status, and microbial modulation in poultry.

Performance indicator	AGPs	AOS supplementation	Observed benefit vs control	Reference
ADG	Increase ADG with dosage 50 mg/kg bacitracin methylene disalicylate	Increase ADG with dosage 200 mg/kg AOS	Comparable or superior to AGPs	[282, 283]
FCR	Can increase FCR in the starter and finisher phases	Improve FCR when administered under controlled conditions, particularly through in ovo technology	Equal or better efficiency	[283, 284]
Egg yolk	Optimizing egg yolk color	The yellow color is more intense and brighter.	AOS superior	[243, 248]
Gut microbiota composition	Reduces pathogens broadly, including commensals	Selective enrichment of *Lactobacillus, Bifidobacterium*	AOS preserves microbial diversity	[315, 316]
Immune markers (IL-6 and sIgA)	Moderate upregulation, often non-targeted	Strong targeted upregulation, enhanced mucosal defense	AOS leads to better immune modulation	[317, 318]
Mortality Rate reduction	1%–2% reduction in mortality	2%–3% reduction, especially under pathogen challenge	AOS as effective or better	[33, 318]
Biofilm inhibition and QS blockade	Not applicable (direct bactericidal mode)	Inhibits quorum-sensing, biofilm disruption	Unique mechanism not found in AGPs	[280, 281]

AGP = Antibiotic growth promoter, AOS = Alginate oligosaccharides, ADG = Average daily gain, FCR = Feed conversion ratio, IL-6 = Interleukin-6, QS = Quorum-sensing, SCFA = Short-chain fatty acids, sIgA = Secretory immunoglobulin A. Comparisons are based on findings from *in vivo* poultry studies conducted under experimental and commercial conditions. Outcomes may vary depending on bird age, diet composition, inclusion level, and management practices.

### Multi-omics approaches to elucidate AOS–microbiome interactions

Advances in metagenomics provide powerful tools for deciphering the genetic composition and functional potential of poultry gut microbiota. The chicken cecum harbors a highly diverse microbial ecosystem dominated by members of the phyla *Firmicutes* and *Bacteroidetes*, which play central roles in nutrient digestion, immune modulation, and resistance to enteric pathogens such as *Campylobacter jejuni* [319]. Complementary to this, metabolomics enables the characterization of microbial-derived metabolites, offering insight into functional outcomes of microbial activity. Notably, the fermentation of oligosaccharides leads to the production of SCFAs, which are critical for gut integrity, host energy metabolism, and regulation of inflammatory responses [320].

The integrated application of metagenomics and metabolomics offers a comprehensive framework for understanding how dietary interventions reshape gut microbial communities and metabolic pathways. This multi-omics approach can identify key microbial taxa, functional genes, and metabolic biomarkers associated with beneficial outcomes, thereby enabling the design of precision nutrition strategies to enhance gut health and growth performance in poultry [321]. Recent evidence suggests that AOS supplementation increases SCFA production, which in turn favorably modulates gut microbiota composition and promotes beneficial bacterial populations [322, 323]. Such insights are instrumental in unraveling the mechanisms underlying AOS functionality and optimizing their use in poultry nutrition.

### Synergistic feed additive strategies

The synergistic integration of AOS with other functional feed additives represents a promising avenue for advancing poultry nutrition. AOS can enhance gut health and nutrient absorption by selectively stimulating beneficial microbial populations, particularly when combined with prebiotics and probiotics such as *Lactobacillus* spp., which produce organic acids and antimicrobial compounds that inhibit pathogenic bacteria [298].

Similarly, dietary enzymes can complement AOS by improving the digestibility of complex feed components, thereby enhancing nutrient utilization and improving performance indicators such as ADG and FCR [324]. Phytogenic feed additives, recognized for their antioxidant, antimicrobial, and immunomodulatory properties, may further augment the benefits of AOS by supporting microbial balance and host immune function [325]. Collectively, these combinatorial strategies offer a viable pathway for improving broiler performance while reducing reliance on AGPs.

### Research gaps and methodological challenges

Despite encouraging findings, several critical research gaps must be addressed to support the broader adoption of AOS, particularly those derived from tropical seaweeds. Long-term, large-scale trials evaluating the sustained effects of tropical AOS on poultry health, productivity, and welfare remain limited. This lack of extended evaluations constrains understanding of their long-term efficacy and consistency under commercial production conditions.

In addition, there is a notable absence of standardized extraction and depolymerization protocols for feed-grade AOS. Variability in production methods, including hydrolytic and enzymatic processes, can significantly influence the physicochemical properties and biological efficacy of AOS [29]. Establishing standardized, reproducible processing techniques is essential to ensure consistent quality, safety, and regulatory compliance.

### Safety, dose optimization, and breed-specific responses

Comprehensive toxicological and residue studies are required to confirm the safety of AOS for human consumption through poultry products. Given their marine origin, AOS may be subject to bioaccumulation of environmental contaminants, necessitating rigorous safety assessments to protect public health [221].

Furthermore, systematic dose–response and bioavailability studies are needed, particularly in tropical poultry breeds. Current literature often extrapolates findings from temperate breeds, overlooking potential differences in metabolic rates, environmental stress tolerance, and nutritional requirements [259]. Targeted research focusing on breed-specific responses would enable more precise dosing strategies and maximize functional benefits.

### Economic and sustainability assessments

Finally, comprehensive economic and sustainability analyses comparing AOS with imported AGPs are largely lacking. While AOS present a promising natural alternative, their widespread adoption depends on clear evidence of cost-effectiveness and scalability [326]. Sustainability assessments should extend beyond environmental metrics to include economic feasibility, supply chain resilience, and socio-economic benefits, particularly in regions with abundant tropical seaweed resources.

Addressing these research priorities will be essential for translating the promising biological potential of AOS into practical, economically viable, and sustainable solutions for modern poultry production systems.

## CONCLUSION

This review comprehensively consolidates current evidence demonstrating that AOS, particularly those derived from tropical brown seaweeds, represent a scientifically credible and functionally effective alternative to AGPs in poultry nutrition. Across in vivo and in vitro studies, AOS consistently improved gut morphology, enhanced microbial balance by promoting beneficial genera such as *Lactobacillus* and *Bifidobacterium*, and increased SCFA production, notably butyrate. These gut-mediated effects translated into measurable improvements in growth performance, FCR, nutrient digestibility, immune competence, antioxidant status, egg production parameters, shell quality, and yolk pigmentation. Importantly, AOS demonstrated comparable or superior performance outcomes to conventional AGPs, while avoiding risks associated with antimicrobial resistance, residue accumulation, and microbiota disruption.

From a practical standpoint, AOS offer multiple advantages for modern poultry systems transitioning toward antibiotic-free production. Their prebiotic, immunomodulatory, antioxidant, and gut-barrier-enhancing properties allow AOS to simultaneously support productivity, animal welfare, and food safety. The compatibility of AOS with pelleted feeds (especially when encapsulated), their synergy with probiotics, enzymes, and phytogenic additives, and their ability to enhance mineral bioavailability and egg quality make them particularly attractive for both broiler and layer operations. Moreover, tropical seaweed-derived AOS provide regionally available, renewable feed resources that may reduce dependency on imported additives, improve feed security, and lower long-term production costs in tropical and subtropical regions.

A major strength of this review lies in its integrative perspective, linking chemistry, physicochemical behavior, gut metabolism, microbiota modulation, immune responses, performance outcomes, and sustainability considerations into a unified framework. The reviewed literature collectively supports a mechanistic understanding of AOS action that parallels and, in several aspects, surpasses AGPs—particularly through QS inhibition, biofilm disruption, selective microbiota enrichment, and SCFA-mediated host responses. Additionally, growing evidence from both poultry and other animal systems (aquaculture and livestock) strengthens the translational relevance of AOS across species.

Despite promising findings, several limitations remain. Long-term, large-scale commercial trials evaluating tropical AOS under diverse production environments are limited. Variability in seaweed species, extraction methods, DP, M/G ratio, and inclusion levels complicates cross-study comparisons and hinders standardization. Furthermore, direct dose–response, bioavailability, and metabolism studies in poultry—especially in tropical breeds—are scarce, with many conclusions extrapolated from mammalian models. Comprehensive toxicological and residue assessments relevant to human food safety are also insufficiently addressed in the current literature. Finally, robust techno-economic and life-cycle sustainability analyses comparing AOS with AGPs and other alternatives remain limited.

Future research should prioritize (i) standardized extraction and depolymerization protocols for feed-grade AOS, (ii) long-term and multi-location poultry trials under commercial conditions, (iii) integrated multi-omics approaches (metagenomics–metabolomics) to elucidate host–microbiome–AOS interactions, (iv) dose optimization and breed-specific response studies, and (v) comprehensive safety, residue, economic, and sustainability evaluations. Exploring synergistic formulations combining AOS with probiotics, enzymes, or phytogenics also represents a promising avenue to maximize functional efficacy while minimizing feed costs.

In conclusion, AOS derived from tropical seaweeds emerge as a strategic, multifunctional, and sustainable feed additive capable of replacing AGPs in poultry production. By enhancing gut health, immune resilience, antioxidant capacity, and production efficiency without contributing to antimicrobial resistance, AOS align strongly with global priorities for sustainable, safe, and responsible animal agriculture. With targeted research addressing existing gaps, tropical AOS have the potential to transition from a promising alternative to a mainstream functional ingredient in next-generation poultry nutrition systems.

## AUTHORS’ CONTRIBUTIONS

SP: Drafted the manuscript. MEM: Revised and edited the manuscript. YR: Drafted and critically revised the manuscript. YLD: Drafted and finalized the manuscript. All authors have read and approved the final version of the manuscript.
